# Feature selection with interactions in logistic regression models using multivariate synergies for a GWAS application

**DOI:** 10.1186/s12864-018-4552-x

**Published:** 2018-03-21

**Authors:** Easton Li Xu, Xiaoning Qian, Qilian Yu, Han Zhang, Shuguang Cui

**Affiliations:** 1 Department of Electrical Engineering and Computer Science, University of Michigan, Ann Arbor, 48109 MI USA; 2 School of Science and Engineering, Chinese University of Hong Kong, Shenzhen, Guangdong, 518172 China; 3 Department of Electrical and Computer Engineering, Texas A&M University, College Station, 77843 TX USA; 4 Department of Electrical and Computer Engineering, University of California, Davis, 95616 CA USA

**Keywords:** Genotype-phenotype association, Feature selection, Genome-wide association study, Synergistic interaction, Mutual information

## Abstract

**Background:**

Genotype-phenotype association has been one of the long-standing problems in bioinformatics. Identifying both the marginal and epistatic effects among genetic markers, such as Single Nucleotide Polymorphisms (SNPs), has been extensively integrated in Genome-Wide Association Studies (GWAS) to help derive “causal” genetic risk factors and their interactions, which play critical roles in life and disease systems. Identifying “synergistic” interactions with respect to the outcome of interest can help accurate phenotypic prediction and understand the underlying mechanism of system behavior. Many statistical measures for estimating synergistic interactions have been proposed in the literature for such a purpose. However, except for empirical performance, there is still no theoretical analysis on the power and limitation of these synergistic interaction measures.

**Results:**

In this paper, it is shown that the existing information-theoretic multivariate synergy depends on a small subset of the interaction parameters in the model, sometimes on only one interaction parameter. In addition, an adjusted version of multivariate synergy is proposed as a new measure to estimate the interactive effects, with experiments conducted over both simulated data sets and a real-world GWAS data set to show the effectiveness.

**Conclusions:**

We provide rigorous theoretical analysis and empirical evidence on why the information-theoretic multivariate synergy helps with identifying genetic risk factors via synergistic interactions. We further establish the rigorous sample complexity analysis on detecting interactive effects, confirmed by both simulated and real-world data sets.

**Electronic supplementary material:**

The online version of this article (10.1186/s12864-018-4552-x) contains supplementary material, which is available to authorized users.

## Background

With the outburst of high-throughput omics data [1–7], there is a pressing need for big data analytics to develop statistical learning algorithms to derive reproducible research findings from extremely high-dimensional data, such that we can better understand complex life and disease systems. Among many analytic problems for big biomedical data, understanding genotype-phenotype relationships is one of the most critical problems to help identify “causal” risk factors and/or biomarkers, further develop accurate phenotypic prediction models, and derive effective therapeutic strategies. In statistical learning, risk factor or biomarker identification problems can be formulated as feature selection or feature screening [8–10] to identify a subset of profiled variables or features that are significantly associated with the system behavior of interest in a statistical sense. Mathematically, given a set of *d* profiled variables, denoted by *X*_1_,*X*_2_,…,*X*_*d*_, we search for a subset of them that are statistically associated (based on *N* sample measurements) with the outcome variable *Y*, which denotes certain systems behavior, such as disease status and treatment response in biomedicine.

Due to the extremely high dimension in modern big data applications, most of the existing feature selection approaches focus on univariate analysis to screen features based on the estimated “individual” or “marginal” effects on the outcome of interest, for example, when looking for genetic risk factors from Single Nucleotide Polymorphisms (SNPs) in many Genome-Wide Association Studies (GWAS) [11]. However, these analyses focusing on individual effects may not be sufficient as real-world systems often manifest complex behaviors arising from highly coordinated interactions among systems components [11–15]. For example, many complex diseases, such as cancer and diabetes, are conjectured to have complicated underlying disease mechanisms, which are neither static nor linear [12–20]. Multiple candidate risk factors, either genetic or environmental, along with their interactions have been considered to play critical roles in triggering and determining the development of diseases [12–20]. Identifying interactive effects among profiled variables not only helps more accurate identification of critical risk factors or biomarkers for outcome prediction, but also helps reveal functional interactions and understand aberrant system changes that are specifically related to the outcome for effective system intervention.

To find important features considering interactive effects, one possible solution is to derive a full Logistic Regression model that incorporates the interactive effects as feature multiplication terms [21]. However, the model complexity can increase exponentially and hence requires a large number of samples to generate reproducible results. Even with the sparse regularization penalty [21], model learning can be computationally expensive when considering interaction terms. Recently, in [22], the authors studied the pairwise interaction in logistic regression models, and establish a rigorous theoretical analysis about how to detect all pairwise interactions. However, they can only deal with the cases when all profiled variables are uniformly distributed, and all pairwise interactions form an acyclic interaction graph.

Due to the prohibitive sample complexity and computational cost when considering the full model with different orders of interactions, most of the existing biomarker identification approaches take a two-step procedure: 1) First, some heuristic measures based on correlation, mutual information, or simplified regression models, are adopted to estimate the statistical association among pairs of features and the outcome [13–20, 23, 24]; 2) Then, some optimization algorithms including greedy ranking algorithms [18–20, 23, 25, 26] are implemented to select “important” features based on various criteria. Due to different possible ad-hoc choices in these methods, it is quite vague which essential information or interaction among features can be captured. The existing literature mostly provides only empirical performance evaluation of these methods without solid theoretical guarantees.

The primary goal of this work is to establish rigorous mathematical theories for feature screening and selection approaches with the consideration of interactive effects under a specific system model based on logistic regression [9, 10], which has been arguably the most popular model for biomarker identification and phenotypic classification, for example, in GWAS. We study the definitions of mutual-information-based synergistic effect measures and try to understand why these measures work under specific model assumptions. We specifically look for interactive effects that are contributing multiplication terms among variables in logistic regression, considered as “cooperative interactions”. We derive a family of interactive measures that can provide accurate detection of such cooperative interactions. We theoretically prove that such interactive measures can indeed be approximately written as quadratic functions of the parameters of the cooperative interactions in logistic regression. In addition, we provide a rigorous theoretical sample complexity analysis on such interactive measures. The two-step procedure with these information-theoretic synergistic interaction measures can accurately and robustly identify risk factors with interactive effects without learning the expensive full logistic regression model. Finally, we apply our results in both simulated data sets and a real-world GWAS data set to demonstrate the effectiveness of these information theoretic measures.

## Methods

### System model

Consider *d* independent binary profiled variables *X*_1_,*X*_2_,…,*X*_*d*_ and a binary outcome variable *Y*. The profiled variables are assumed to have the probability distribution Pr(*X*_*i*_=+1)=*p*_*i*_ and Pr(*X*_*i*_=−1)=*q*_*i*_ with *p*_*i*_,*q*_*i*_>0, *p*_*i*_+*q*_*i*_=1 for 1≤*i*≤*d*, and the conditional probability of the outcome variable *Y* is assumed to take the following form:

1 $$ \begin{aligned} \Pr(Y&=1|X_{1},X_{2},\ldots,X_{d})\\ &=\sigma\left(\beta_{\emptyset}+\sum_{\emptyset \subset S\subseteq\{1,2,\ldots,d\}}\beta_{S}\prod_{i\in S} X_{i}\right),\\ \end{aligned}  $$

2 $$ \begin{aligned} \Pr(Y&=-1|X_{1},X_{2},\ldots,X_{d})\\ &=1-\Pr(Y=1|X_{1},X_{2},\ldots,X_{d}), \end{aligned}  $$

where *σ*(*x*):=1/(1+*e*^−*x*^) is the sigmoid function and {*β*_*S*_:*S*⊆{1,2,…,*d*}} is a family of real parameters. For any subset *S* of {1,2,…,*d*}, parameter *β*_*S*_ measures the amount of the cooperative interaction among the variables *X*_*i*_’s (*i*∈*S*). We call this model as the “full” model. Assume that all parameters *β*_*S*_ are bounded, i.e., |*β*_*S*_|<*C* for all *S*⊆{1,2,…,*d*}. It is a highly generic model based on the classical logistic regression model, since it incorporates the cooperative interaction of any subset of profiled variables *X*_1_,*X*_2_,…,*X*_*d*_. We can estimate the cooperative interactions among candidates *X*_*i*_’s and *Y* via the help of multivariate information measures, which are suggested to quantify the correlation among two or more random variables. Such measures include multivariate mutual information [27–32], Pearson’s correlation coefficients [33], and maximal information coefficient [34]. Multivariate mutual information, an information theoretical [35, 36] tool, has a variety of definitions, such as multivariate synergy [13, 14, 37], McGill’s mutual information [27], Watanabe’s total correlation [28], Gács-Körner common information [29], Han’s dual total correlation [30], and Wyner’s common information [31]. In [32], the authors compared the mathematical and information-theoretical properties among many existing multivariate mutual information measures and suggested a new one inspired by multi-terminal secret-key agreement [38].

In this paper, we mainly focus on the multivariate synergy, first suggested in [37] (where a different notation “ *RS*_*N*|*N*−1_” was used) and recently proposed for interaction and association studies in bioinformatics by Anastassiou [13]. Precisely, for any *n* random variables *Z*_1_,*Z*_2_,…,*Z*_*n*_, the multivariate synergy  of these variables is defined to be



where *H* is the Shannon entropy [35, 36]. Notice that when *n*=2, the multivariate synergy  is in fact the mutual information of *Z*_1_ and *Z*_2_ [35], a measure of the dependence between *Z*_1_ and *Z*_2_ in information theory.

In the following, we first connect the defined multivariate synergies with cooperative interactions manifested as the coefficients of the corresponding interaction terms in the full logistic regression model.

The main theoretical result that we establish is to show why such a multivariate synergy can help risk factor identification with interactions. Based on the connection between multivariate synergy and the interaction terms in the logistic regression model, we further derive the sample complexity for accurate interaction estimation.

### Estimation of interaction parameters by multivariate synergies

We first establish the main theorem, which shows that for any subset *S* of {1,2,…,*d*}, the multivariate synergy of $X_{S}\triangleq \{X_{i}: i\in S\}$ and *Y* is approaching a quadratic function over parameter *β*_*S*_ of the cooperative interaction corresponding to *S*.

#### **Theorem 1**

For any subset *S*⊆{1,2,…,*d*},



#### *Proof*

See the proof in Additional file 1. □

The above theorem shows that the multivariate synergy depends only on the interaction parameters *β*_*G*_ for *G*⊇*S* approximately, when *C* is small enough. For the special case when all profiled variables *X*_*i*_’s are uniformly distributed, the theorem has a cleaner form as follows.

#### **Corollary 2**

Assume that each profiled variable *X*_*i*_ is uniformly distributed. For any set *S*⊆{1,2,…,*d*}, we have 

#### *Proof*

It quickly follows from Theorem 1 with *p*_*i*_=*q*_*i*_=1/2 for *i*∈*G*∖*S*. □

From this corollary, it is clear that the multivariate synergy mainly depends on *β*_*S*_ when *C* is small enough. Hence, estimating the multivariate synergy can help identify interactions without inferring the full logistic regression model.

For the interactions of the highest order, we have another result in a clean form.

#### **Corollary 3**

Assume that there is no interaction of orders higher than *m*, i.e., *β*_*G*_=0 if |*G*|>*m*. For any set *S*⊆{1,2,…,*d*} with order *m*, we have



#### *Proof*

This follows from Theorem 1 with *β*_*G*_=0 for *G*⊃*S*. □

This result tells us that the highest-order multivariate synergy mainly depends on *β*_*S*_ when *C* is small enough. This indeed guarantees that when the sample size is large enough, we can correctly estimate the highest-order interactions in logistic regression without actually learning the full model.

Based on the above results, we find that the multivariate synergy has a monotonic relationship with the magnitude of the interactive effects in the full logistic regression model, which explains the past empirical results showing that they indeed work in GWAS. In addition, we also notice that such a monotonic relationship can be interfered by the common factor $\frac {1}{8}\prod _{i\in S} 4p_{i}q_{i}$, dependent on the distributions of *X*_*i*_ in *S*. To alleviate such interference, we propose an **adjusted multivariate synergy**, which directly reflects the interactive effect in the logistic regression model with the normalization to adjust for the interference:

#### **Definition 4**

**Adjusted Multivariate Synergy:**
3


In the experiments, we will demonstrate that this new proposed measure can accurately and robustly identify interactions from both simulated and real-world GWAS data.

### Number of samples needed for estimation

In this section, we provide the lower bound of the number of samples that we need to ensure the small estimation error of the multivariate synergy. For any variables *Z*_1_,*Z*_2_,…,*Z*_*t*_ on {1,−1}, the plug-in estimate $\widehat {H}_{N}(Z_{1},Z_{2},\ldots,Z_{t})$ of the entropy *H*(*Z*_1_,*Z*_2_,…,*Z*_*t*_) is defined as [39]:

$$\begin{array}{*{20}l} &\widehat{H}_{N}(Z_{1},Z_{2},\ldots,Z_{t})\\ &=-\sum_{z_{1},z_{2},\ldots,z_{t}\in \{1,-1\}} \hat{p}_{z_{1},z_{2},\ldots,z_{t}}\log \hat{p}_{z_{1},z_{2},\ldots,z_{t}}, \end{array} $$

where $\hat {p}_{z_{1},z_{2},\ldots,z_{t}}$ is the empirical probability of {*Z*_1_=*z*_1_,*Z*_2_=*z*_2_,…,*Z*_*t*_=*z*_*t*_}. By Lemma 6 in Additional file 1, the plug-in estimate  of  can be written as



Then we establish the following theorem about the sample complexity for estimation of .

#### **Theorem 5**

For 0<*ε*,*δ*<1, choose

$$N\ge \frac{e^{2}}{(e-2)^{2}}\widetilde{N}(\varepsilon,\delta)\left[\log \widetilde{N}(\varepsilon,\delta)\right]^{2}, $$

where *e* is the base of the natural logarithm and

$$\widetilde{N}(\varepsilon,\delta)=\frac{2^{2|S|+3}}{\delta^{2}}\log \frac{\max\left\{2^{|S|+1},6\right\}}{\varepsilon}, $$

then we have



#### *Proof*

See the proof in Additional file 1. □

We note that the sample complexity is exponential over the interaction order to detect.

## Results

With these established theoretical results, we now empirically test the effectiveness of the information-theoretic synergistic interaction measures, including our proposed adjusted multivariate synergy defined in (3).

### Simulated data

We randomly generate 1000 logistic regression models. Each model contains 50 features. We randomly choose 3 features and 3 interacting pairs as contributing terms to the outcome for this model, and randomly assign a parameter drawn from a uniform distribution over [ 1,2] quantifying the effect size for each of these features and pairs. For each logistic regression model, we generate random training sets of 500, 1000, 1500, 2000, 2500, and 3000 samples. Each training sample consists of an observation of each covariate *X*_*i*_ drawn from a two-point distribution (Pr(*X*_*i*_=1)=*p*_*i*_ and Pr(*X*_*i*_=−1)=1−*p*_*i*_), for 1≤*i*≤50, and a binary outcome from the conditional distributions (1), (2), where *p*_*i*_ is randomly drawn from a uniform distribution over {0.1,0.2,0.3,0.4,0.5,0.6,0.7,0.8,0.9}. With these randomly generated training samples of different sizes, we detect the three chosen pairs in the logistic regression model using six different information-theoretic measures: 1) multivariate synergy [13, 37], 2) adjusted multivariate synergy, 3) Schneidman’s normalized synergy [40], 4) Ignac’s normalized mutual information [41], 5) Watabene’s total correlation [28], and 6) Han’s dual total correlation [30].

**Fig. 1:**
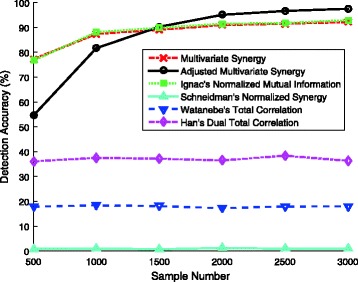
Detection accuracies of interactive effects by the methods based on six information-theoretic measures (with 50 features)

For each measure, we consider the three pairs with the largest estimated values of this measure as the interacting pairs, and evaluate the detection correctness. Figure 1 shows that the methods based on multivariate synergy, adjusted multivariate synergy, and Ignac’s normalized mutual information highly outperform the other three methods based on Watabene’s total correlation, Han’s dual total correlation, and Schneidman’s normalized synergy. Furthermore, the algorithm based on the multivariate synergy or Ignac’s normalized mutual information performs the best when the number of samples is at most 1000, while the method of ranking the adjusted multivariate synergies achieves a roughly 5% higher accuracy than that of ranking the multivariate synergies or Schneidman’s normalized synergies when the number of the samples are 1500, 2000, 2500, and 3000. By the independent two-sample t-test, the corresponding *p*-value is less than 10^−5^, which shows the statistically significant difference between the detection accuracies. The adjusted multivariate synergy is directly related to the interaction parameter according to Corollary 3. Thus it can well capture the interactive effect via the normalization. It needs a little more samples to get a relatively accurate estimate (both its numerator and denominator need to be estimated) compared to multivariate synergy. We conjecture that the combination of these two measures probably could serve as a more useful tool for interaction detection. The other methods based on Schneidman’s normalized synergy, Watabene’s total correlation, or Han’s dual total correlation have inferior performance when identifying the interactions, since these measures have no clear relationships with the interaction parameters in logistic regression models.

We further study the relationship between the sample number and the detection accuracy of the interactive effects. It is observed that the curves obtained by both the multivariate synergy and the adjusted multivariate synergy fit very well with a logarithmic relation: i.e.,

$$\textrm{sample number \textit{N}} \propto \log(1/\textrm{detection error rate \(\varepsilon\)}), $$

closely matching the derived theoretical bound on sample complexity in Theorem 5.

**Fig. 2:**
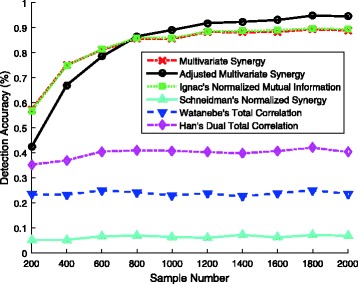
Detection accuracies of the methods based on six information-theoretic measures (with 20 features)

Here we remark that the same conclusion can be drawn with different settings on the number of features in the model. We also generate 1000 logistic regression models, each of which contains 20 features. In each model, we randomly generate 200, 400, 600, 800, 1000, 1200, 1400, 1600, 1800, and 2000 samples by the same way as in the previous simulation. Figure 2 illustrates the prediction accuracies of the models based on the six aforementioned theoretic measures. The same trends as discussed earlier can be observed in the figure.

**Fig. 3:**
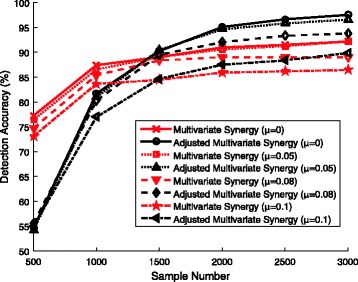
Detection accuracies of the interactive effects by the methods based on multivariate synergy and adjusted multivariate synergy with different *μ*

Although we derive the theoretical results with the assumption that the features are independent of each other, the multivariate synergy and the adjusted multivariate synergy can still serve as good measures of interactions for the cases when the features are weakly dependent in practice. We further simulate such weakly dependent cases to empirically evaluate their interaction detection performance. In each simulated full logistic regression model, we first randomly choose *K* from a uniform distribution on {1,−1}, and then each covariate *X*_*i*_ is drawn from a conditional probability Pr(*X*_*i*_=1|*K*)=*p*_*i*_+*μK* and Pr(*X*_*i*_=−1|*K*)=*q*_*i*_−*μK*, for 1≤*i*≤50, with *μ* controlling the dependency among covariates. Here, *p*_*i*_ is randomly chosen from a uniform distribution over {0.1,0.2,0.3,0.4,0.5,0.6,0.7,0.8,0.9} as in the previous simulations. The output *Y* is generated by the conditional probability (1) and (2). Straightforward calculation shows that each pair of features are dependent with covariance 4*μ*^2^ and coefficient correlation *ρ*∈[4*μ*^2^,100*μ*^2^/9], when *μ*≠0. Figure 3 plots the detection accuracies of the methods based on multivariate synergy or adjusted multivariate synergy with different *μ* (*μ*=0, 0.05, 0.08, or 0.1). The trends are clear that these measures still can help accurately identify the interactions even when all pairs of features are weakly dependent, especially when *μ* is small (< 0.1). The relationship between the sample complexity and detection error rate still follows the derived logarithmic relationship with weakly dependent features.

### Real-world GWAS data

Type 1 Diabetes (T1D), previously known as Insulin-Dependent Diabetes Mellitus (IDDM), is an autoimmune disease resulting from the deficiency of insulin. This disease is conjectured to be caused by both genetic and environmental factors and has attracted tremendous research interests, especially in detecting pairwise or high-order genome-wide interactions for T1D [42–46]. Here, we apply our proposed adjusted multivariate synergy to the case-control data extracted from the Wellcome Trust Case Control Consortium (WTCCC) [47]. The WTCCC T1D data set includes 2000 case samples and 1500 control samples, each of which contains around 500,000 SNPs. In [42], the BOOST method, a two-stage (screening and testing) search method, selects the pairs without significant main effects and with significant interactions. They listed all 91 such pairs in Table S6 of [42] (referred as “Table W” in this paper), each of which satisfies that the genome distance between the two SNPs’ chromosomal positions is at least 1Mb. To make the comparison between our method and theirs, we pick 73 SNPs mentioned in the table, and run our algorithm on the part of the data containing the information related to these SNPs. We estimate the adjusted multivariate synergy for each pair of these SNPs. The pairs with the 15 largest estimates are shown in Table 1.

**Table 1:** The top 15 pairs with the largest adjusted multivariate synergy estimates

SNP A	SNP B	Adjusted multivariate synergy estimates
rs2516486	rs6919798	0.4558286
rs2516486	rs9276448	0.4544231
rs2516486	rs5014418	0.4513707
rs2894180	rs5014418	0.4274221
rs2894180	rs9276448	0.4218615
rs2894180	rs6919798	0.4181264
rs2516486	rs9276299	0.3801617
rs2516486	rs9276227	0.3781777
rs707937	rs6919798	0.3558587
rs3095250	rs5014418	0.3259175
rs3095250	rs9276448	0.3182534
rs3873385	rs5014418	0.3153821
rs3873385	rs9276448	0.3150227
rs2894180	rs427037	0.3145728
rs2853934	rs9276448	0.3091304

Fisher’s exact test [48] has been carried out for enrichment analysis [49] of the SNPs ranked on top using our adjusted multivariate synergy estimates and the SNPs in the listed pairs in Table W. Notice that all the 15 interacting pairs in Table 1 are listed in Table W, with a significant *p*-value 1.467×10^−18^. Also, the pairs with the 681 largest estimates selected by our algorithm cover all 91 pairs in Table W, giving a *p*-value 1.044×10^−27^. Further, Table W contains 17 pairs (respectively, 13 pairs) with the lowest PLINK *p*-value [50] (respectively, BOOST *p*-value) 1.100×10^−16^, and they are included in the set of the pairs with the 76 (respectively, 103) largest estimates selected by our algorithm. The corresponding *p*-values in Fisher’s exact tests are 1.678×10^−22^ and 2.268×10^−15^, respectively. These significant *p*-values shows the highly significant overlap between the interacting SNP pairs found by our algorithm and those by BOOST and PLINK.

We further check the biological interpretation of the top ranked SNPs with significant interactions. The associated genes with the SNPs in the top 15 interacting pairs are listed in Table 2. Gene ontology enrichment analysis is implemented via the Gene Ontology Database for Homo sapiens [51]. In Table 3, we list the gene ontology classes with the five smallest *p*-values in terms of their associated cellular components, molecular functions, and biological processes, respectively. Observe that for each root category of the gene ontology terms, at least one of top 5 classes is related to the Major Histocompatibility Complex (MHC) with *p*-values less than 0.05. Note that in the T1D literature [52–55], MHC has already been proved to have strong association with T1D development.

**Table 2:** Associated genes with the SNPs in the top 15 interacting pairs

SNP	Gene Associations
rs2516486	MCCD1, RPL15P4, DASS-161H22.6,
	ATP6V1G2-DDX39B, DDX39B
rs6919798	HLA-DQB2
rs9276448	HLA-DQA2
rs5014418	HLA-DQB2, HLA-DQA2
rs2894180	HCG27, XXbac-BPG299F13.14
rs9276299	HLA-DQB3, HLA-DQA2
rs9276227	HLA-DQB3, HLA-DQA2
rs707937	MSH5, SAPCD1, MSH5-SAPCD1,
	Xbac-BPG32J3.18, VWA7
rs3095250	HCG27,HLA-C
rs3873385	HLA-B, XXbac-BPG248L24.13
rs427037	none
rs2853934	WASF5P, HLA-B, RPL3P2

**Table 3:** Gene ontology enrichment analysis

Ontology	Gene ontology class	*p*-value
Cellular component	1. MHC protein complex	1.32E-06
	2. Integral component of lumenal side of ER membrane	1.52E-06
	3. Lumenal side of ER membrane	1.52E-06
	4. ER to Golgi transport vesicle membrane	2.09E-05
	5. ER to Golgi transport vesicle	7.44E-05
Molecular function	1. Peptide antigen binding	1.50E-03
	2. TAP binding	1.58E-02
	3. MHC class II receptor activity	3.91E-02
	4. Antigen binding	4.95E-01
	5. Peptide binding	6.13E-01
Biological process	1. Interferon-gamma-mediated signaling pathway	3.49E-04
	2. Cellular response to interferon-gamma	3.30E-03
	3. Response to interferon-gamma	6.28E-03
	4. Antigen processing and presentation of endogenous peptide antigen via MHC class I via ER pathway, TAP-independent	9.23E-03
	5. Antigen processing and presentation of endogenous peptide antigen via MHC class I via ER pathway	9.23E-03

**Fig. 4:**
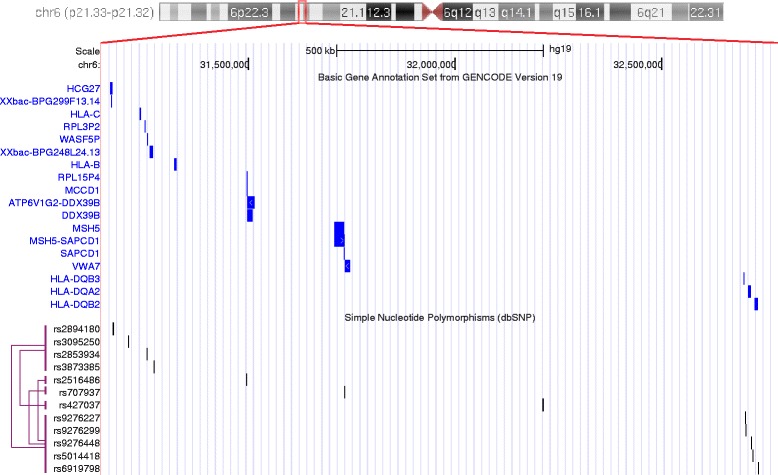
Genome mapping of SNPs

Genome mapping of SNPs is further illustrated in Fig. 4, where we also visualize the identified top interacting SNP pairs given in Table 1. We notice that the corresponding genes to which these SNPs are mapped to are interacting with each other. For example, the interaction between MSH5 and the genes encoding MHC class II molecules has been reported in [56, 57], conjecturing that they play synergistic roles in T1D development.

## Discussions

As discussed in previous simulation and real-world data experiments, multivariate synergy measures are effective in identifying interactions among candidate risk factors for genotype-phenotype association studies. This is expected from our derived theoretical connection between synergy and interaction parameters in logistic regression modeling of genotype-phenotype relationships. On the other hand, accurate and reproducible identification of interactive factors requires that the number of samples grows exponentially with the order of interactions to detect. Based on the given number of samples, identified interactions should be thoroughly validated with caution.

## Conclusions

In this paper, we study why the multivariate synergy can serve as a measure to quantify the interaction among multiple factors for feature selection with interactions. We further have established the theoretical analysis on sample complexity, which is general for feature selection when considering interactions. For risk factor identification in GWAS, when the genotype-phenotype association is modeled by logistic regression, we show that the multivariate synergies have a close relationship with the corresponding multiplication parameters capturing the interactive effects among features. Based on such derived relationships, we have proposed an “adjusted multivariate synergy” as a new interaction measure. The experiments showed the adjusted multivariate synergy achieves an excellent empirical performance in risk factor identification with interactions over both simulated and real-world T1D GWAS data.

## Additional file

Additional file 1 Some useful lemmas and the proofs of Theorems 1 and 5. (PDF 89 kb)

## Abbreviations

GWAS: Genome-wide association studies
IDDM: Insulin-dependent diabetes mellitus
MHC: Major histocompatibility complex
SNP: Single nucleotide polymorphisms
T1D: Type 1 diabetes
WTCCC: Wellcome trust case control consortium
