# Supplementary material for: Feature selection with interactions in logistic regression models using multivariate synergies for a GWAS application
Source: BMC Genomics. 2018 Mar 21;19(Suppl 4):170. doi: 10.1186/s12864-018-4552-x (PMC5872388; doi:10.1186/s12864-018-4552-x)
Supplement: Supplementary file 1 — Some useful lemmas and the proofs of Theorems 1 and 5. (PDF 89 kb) [file 12864_2018_4552_MOESM1_ESM.pdf]

Useful Lemmas

We first introduce the following lemmas that are useful to prove Theorems 1 and 5.

Lemma 6 *For any subset $S \subseteq \{1, 2, \ldots, d\}$,*

$$\begin{aligned}\mathscr{S}(X_S; Y) &= \sum_{\emptyset \subseteq T \subseteq S} (-1)^{|S|+|T|+1} H(Y|X_T) \\ &= \sum_{\emptyset \subseteq T \subseteq S} (-1)^{|S|+|T|+1} H(X_T, Y) \\ &\quad + \mathbf{1}_{|S|=1} H(X_S),\end{aligned}$$

where $\mathbf{1}$ is the indicator function.

Proof By the definition of multivariate synergy of $(|S|+1)$ variables, including $\{X_i : i \in S\}$ and Y, we have

$$\begin{aligned}\mathscr{S}(X_S; Y) = &\sum_{\emptyset \subset T \subseteq S} (-1)^{|S|+|T|} H(X_T) \\ &+ \sum_{\emptyset \subseteq T \subseteq S} (-1)^{|S|+|T|+1} H(X_T, Y) \\ = &\sum_{\emptyset \subseteq T \subseteq S} (-1)^{|S|+|T|+1} H(Y|X_T).\end{aligned}$$

Since $X_1, X_2, \ldots, X_d$ are independent, we have

$$\begin{aligned}\sum_{\emptyset \subset T \subseteq S} (-1)^{|S|+|T|} H(X_T) &= \sum_{\emptyset \subset T \subseteq S} (-1)^{|S|+|T|} \sum_{i \in T} H(X_i) \\ &= \begin{cases} 0 & \text{if } |S| > 1; \\ H(X_S) & \text{if } |S| = 1. \end{cases}\end{aligned}$$

□

Let $h(x) = -x \log x$ for $x > 0$ and $h(0) = 0$; we have the following lemma.

Lemma 7 *Let A, B, C, D be nonnegative numbers, $E = A - B + C - D$, $F = A - B - C + D$, and $G = A + B + C + D > 0$. Let $\zeta = \max\{|\lambda|, |\mu|\}$. We have*

$$\begin{aligned}&h(A\sigma(\lambda+\mu) + B\sigma(-\lambda-\mu) + C\sigma(\lambda-\mu) + D\sigma(-\lambda+\mu)) \\ &+ h(B\sigma(\lambda+\mu) + A\sigma(-\lambda-\mu) + D\sigma(\lambda-\mu) + C\sigma(-\lambda+\mu)) \\ &= G \log 2 + h(G) - \frac{1}{8G}(E\lambda + F\mu)^2 + O(\zeta^4). \qquad (4)\end{aligned}$$

Proof Proved by calculating the first, second, third of the partial derivatives of the left-hand side of (4) with respect to λ and μ. □

Lemma 8 *Let X be a discrete random variable with the probability distribution $\Pr(X = a_i) = p_i$ for $1 \leq i \leq m$, and $\sum_{i=1}^{m} p_i = 1$. We draw N samples $X^{(1)}, X^{(2)}, \ldots, X^{(N)}$ from the distribution of X, and calculate the plug-in estimate of the entropy $H(X)$ as*

$$\widehat{H}_N(X) \triangleq -\sum_{i=1}^{m} \frac{n_i}{N} \log \frac{n_i}{N},$$

where

$$n_i = \sum_{j=1}^{N} \mathbf{1}_{X^{(j)} = a_i}.$$

Then

$$\begin{aligned}&\Pr\left(\left|\widehat{H}_N(X) - \mathbb{E}[\widehat{H}_N(X)]\right| > \varepsilon\right) \\ &\qquad \leq 2 \exp\left(-\frac{1}{2} N (\log N)^{-2} \varepsilon^2\right),\end{aligned}$$

and

$$0 \leq H(X) - \mathbb{E}[\widehat{H}_N(X)] \leq \log\left(1 + \frac{m-1}{N}\right) \leq \frac{m-1}{N}.$$

Proof The proof can be found in [39]. □

Proof of Theorem 1

Let $\mathcal{I} = \{1, 2, \ldots, d\}$. Without loss of generality, we assume that $S = \{1, 2, \ldots, m\}$ with $1 \leq m \leq d$. Consider a binary variable $\widetilde{Y} = -Y$. Then for any $X_1, X_2, \ldots, X_d$, $\widetilde{Y}$ has the conditional probability distribution

$$\begin{aligned}&\Pr(\widetilde{Y} = 1 | X_1, X_2, \ldots, X_d) \\ &\qquad = \sigma\left(-\beta_\emptyset + \sum_{\emptyset \subset T \subseteq \mathcal{I}} (-\beta_T) \prod_{i \in T} X_i\right) \\ &\Pr(\widetilde{Y} = -1 | X_1, X_2, \ldots, X_d) \\ &\qquad = 1 - \Pr(\widetilde{Y} = 1 | X_1, X_2, \ldots, X_d).\end{aligned}$$

Notice that the multivariate synergy of $X_1, X_2, \ldots, X_m$ with respect to $\widetilde{Y}$ is the same as that of $X_1, X_2, \ldots, X_m$ and Y, which implies that if we replace every parameter $\beta \in \{\beta_T : \emptyset \subseteq T \subseteq \mathcal{I}\}$ with its negative $-\beta$ in the Taylor series of $\mathscr{S}(X_S; \widetilde{Y})$, the Taylor series keeps the same. This observation tells us that the Taylor series of $\mathscr{S}(X_S; Y)$ contains only the even-order terms. Also, if we set every parameter $\beta \in \{\beta_T : \emptyset \subseteq T \subseteq \mathcal{I}\}$ to be zero, the conditional probability distribution of Y with respect to $X_1, X_2, \ldots, X_d$ is always uniformly distributed, and thereby $\mathscr{S}(X_S; Y) = 0$. Hence, the constant term in the Taylor series of $\mathscr{S}(X_S; Y)$ is 0.

Now we only need to calculate the coefficients of the quadratic terms in the Taylor series of $\mathscr{S}(X_S;Y)$. For notational simplicity, let $p_{i,1}=p_i$ and $p_{i,-1}=q_i$. We denote $\{X_T=x_T\}$ to be the event $\bigcup_{i\in T}\{X_i=x_i\}$. Then $\Pr(X_T=x_T)=\prod_{i\in T}p_{i,x_i}$. Pick any two parameters

$$\lambda,\mu\in\{\beta_T:\emptyset\subseteq T\subseteq\mathcal{I}\}.$$

After setting all parameters in

$$\bar{\beta}\triangleq\{\beta_T:\emptyset\subseteq T\subseteq\mathcal{I}\}\setminus\{\lambda,\mu\}$$

to be zero, the coefficients of $\lambda^2,\lambda\mu,\mu^2$ remain unchanged.

For any subset $T\subseteq S$ and $x_T\in\{1,-1\}^{|T|}$, we have

$$\begin{aligned}&\Pr(Y=1|X_T=x_T)\big|_{\bar{\beta}=0}\\&\quad=\frac{1}{\Pr(X_T=x_T)}\sum_{x_{\mathcal{I}\setminus T}}\Pr(X_{\mathcal{I}}=x_{\mathcal{I}},Y=1)|_{\bar{\beta}=0}\\&\quad=\sum_{x_{\mathcal{I}\setminus T}}\Pr(X_{\mathcal{I}\setminus T}=x_{\mathcal{I}\setminus T})\Pr(Y=1|X_{\mathcal{I}}=x_{\mathcal{I}})|_{\bar{\beta}=0}.\end{aligned}$$

Notice that for any $x_{\mathcal{I}}$, $\Pr(Y=1|X_{\mathcal{I}}=x_{\mathcal{I}})_{\bar{\beta}=0}$ is $\sigma(\lambda+\mu)$, $\sigma(-\lambda-\mu)$, $\sigma(\lambda-\mu)$, or $\sigma(-\lambda+\mu)$. Then we write

$$\begin{aligned}&\Pr(Y=1|X_T=x_T)\big|_{\bar{\beta}=0}\\&\quad=A_{X_T=x_T}\sigma(\lambda+\mu)+B_{X_T=x_T}\sigma(-\lambda-\mu)\\&\qquad+C_{X_T=x_T}\sigma(\lambda-\mu)+D_{X_T=x_T}\sigma(-\lambda+\mu),\end{aligned}\tag{5}$$

where $A_{X_T=x_T},B_{X_T=x_T},C_{X_T=x_T}$, and $D_{X_T=x_T}$ are the coefficients of

$$\sigma(\lambda+\mu),\sigma(-\lambda-\mu),\sigma(\lambda-\mu),\text{ and }\sigma(-\lambda+\mu),$$

respectively, in $\Pr(Y=1|X_T=x_T)\big|_{\bar{\beta}=0}$. Thus $A_{X_T=x_T}+B_{X_T=x_T}+C_{X_T=x_T}+D_{X_T=x_T}=1$, and

$$\begin{aligned}&\Pr(Y=-1|X_T=x_T)\big|_{\bar{\beta}=0}\\&\quad=B_{X_T=x_T}\sigma(\lambda+\mu)+A_{X_T=x_T}\sigma(-\lambda-\mu)\\&\qquad+D_{X_T=x_T}\sigma(\lambda-\mu)+C_{X_T=x_T}\sigma(-\lambda+\mu).\end{aligned}$$

Let

$$\begin{aligned}E_{X_T=x_T}&=A_{X_T=x_T}-B_{X_T=x_T}+C_{X_T=x_T}-D_{X_T=x_T},\\F_{X_T=x_T}&=A_{X_T=x_T}-B_{X_T=x_T}-C_{X_T=x_T}+D_{X_T=x_T},\end{aligned}$$

for $x_T\in\{1,-1\}^{|T|}$. By Lemma 7, we have

$$\begin{aligned}&h(\Pr(X_T=x_T,Y=1))\big|_{\bar{\beta}=0}\\&+h(\Pr(X_T=x_T,Y=-1))\big|_{\bar{\beta}=0}\\&=\Pr(X_T=x_T)\log 2+h(\Pr(X_T=x_T))\\&\quad-\frac{1}{8}\Pr(X_T=x_T)(E_{X_T=x_T}\lambda+F_{X_T=x_T}\mu)^2+O(C^4).\end{aligned}\tag{6}$$

Let $\Lambda_{X_T=x_T}=E_{X_T=x_T}\lambda+F_{X_T=x_T}\mu$. By summing equation (6) over all x_T, we have

$$\begin{aligned}&H(X_T,Y)\big|_{\bar{\beta}=0}\\&=\sum_{x_T}\Big[\Pr(X_T=x_T)\log 2+h(\Pr(X_T=x_T))\\&\quad-\frac{1}{8}\Pr(X_T=x_T)(E_{X_T=x_T}\lambda+F_{X_T=x_T}\mu)^2\Big]+O(C^4)\\&=\log 2+H(X_T)-\frac{1}{8}\sum_{x_T}\Pr(X_T=x_T)\Lambda^2_{X_T=x_T}+O(C^4).\end{aligned}$$

Then by Lemma 6, we have

$$\begin{aligned}&\mathscr{S}(X_S;Y)\big|_{\bar{\beta}=0}\\&=\sum_{\emptyset\subseteq T\subseteq S}(-1)^{|S|+|T|+1}H(Y|X_T)\big|_{\bar{\beta}=0}\\&=\sum_{\emptyset\subseteq T\subseteq S}(-1)^{|S|+|T|+1}\Big(\log 2\\&\qquad-\frac{1}{8}\sum_{x_T}\Pr(X_T=x_T)\Lambda^2_{X_T=x_T}\Big)+O(C^4)\\&=\frac{1}{8}\sum_{\emptyset\subseteq T\subseteq S}(-1)^{m+|T|}\sum_{x_T}\frac{1}{\Pr(X_T=x_T)}\\&\qquad\cdot\left(\sum_{x_{S\setminus T}}\Pr(X_S=x_S)\Lambda_{X_S=x_S}\right)^2+O(C^4)\\&\overset{(a)}{=}\frac{1}{8}\left[\prod_{i\in S}p_iq_i\right]\left[\sum_{x_S}\left(\prod_{i\in S}x_i\right)\Lambda_{X_S=x_S}\right]^2+O(C^4)\\&=\frac{1}{8}\left[\prod_{i\in S}p_iq_i\right]\left[\lambda\sum_{x_S}\left(\prod_{i\in S}x_i\right)E_{X_S=x_S}\right.\\&\qquad\left.+\mu\sum_{x_S}\left(\prod_{i\in S}x_i\right)F_{X_S=x_S}\right]^2+O(C^4),\end{aligned}\tag{7}$$

where the proof of (a) is in the next section.

Now we discuss the value of $\sum_{x_S}\left(\prod_{i\in S}x_i\right)E_{X_S=X_S}$ in the following three cases.

Case 1: λ is β_S. For $x_S \in \{1,-1\}^{|S|}$, we have

$$\Pr(Y=1|X_S=x_S)\big|_{\mu=0,\bar{\beta}=0}=\sigma\left(\lambda\prod_{i\in S}x_i\right).$$

By (5), we have

$$\begin{aligned}&\Pr(Y=1|X_S=x_S)\big|_{\mu=0,\bar{\beta}=0}\\&\quad=(A_{X_S=x_S}+C_{X_S=x_S})\sigma(\lambda)\\&\qquad+(B_{X_S=x_S}+D_{X_S=x_S})\sigma(-\lambda).\end{aligned}$$

Thus,

$$\begin{aligned}E_{X_S=x_S}&=A_{X_S=x_S}-B_{X_S=x_S}+C_{X_S=x_S}-D_{X_S=x_S}\\&=\prod_{i\in S}x_i,\end{aligned}$$

and hence

$$\sum_{x_S}\left(\prod_{i\in S}x_i\right)E_{X_S=x_S}=\sum_{x_S}1=2^m.$$

Case 2: λ is β_V with $S\not\subseteq V$. There exists $k\in S\setminus V$, and then $\Pr(Y=1|X_S=x_S)\big|_{\mu=0,\bar{\beta}=0}$ is independent of X_k. For notational simplicity, assume $k=m$, and let $\bar{S}=S\setminus\{m\}$. Hence,

$$\begin{aligned}&\Pr(Y=1|X_{\bar{S}}=x_{\bar{S}},X_m=1)\big|_{\mu=0,\bar{\beta}=0}\\&=\Pr(Y=1|X_{\bar{S}}=x_{\bar{S}},X_m=-1)\big|_{\mu=0,\bar{\beta}=0}.\end{aligned}$$

By (5), we have

$$\begin{aligned}&(A_{X_{\bar{S}}=x_{\bar{S}},X_m=1}+C_{X_{\bar{S}}=x_{\bar{S}},X_m=1})\sigma(\lambda)\\&\quad+(B_{X_{\bar{S}}=x_{\bar{S}},X_m=1}+D_{X_{\bar{S}}=x_{\bar{S}},X_m=1})\sigma(-\lambda)\\&=(A_{X_{\bar{S}}=x_{\bar{S}},X_m=-1}+C_{X_{\bar{S}}=x_{\bar{S}},X_m=-1})\sigma(\lambda)\\&\quad+(B_{X_{\bar{S}}=x_{\bar{S}},X_m=-1}+D_{X_{\bar{S}}=x_{\bar{S}},X_m=-1}))\sigma(-\lambda).\end{aligned}$$

Thus,

$$E_{X_{\bar{S}}=x_{\bar{S}},X_m=1}=E_{X_{\bar{S}}=x_{\bar{S}},X_m=-1},$$

and

$$\begin{aligned}\sum_{x_S}\left(\prod_{i\in S}x_i\right)E_{X_S=x_S}&=\sum_{x_{\bar{S}}}\left(\prod_{i\in\bar{S}}x_i\right)\\&\quad\cdot(E_{X_{\bar{S}}=x_{\bar{S}},X_m=1}-E_{X_{\bar{S}}=x_{\bar{S}},X_m=-1})=0.\end{aligned}$$

Case 3: λ is β_V with $V\supset S$. For notational simplicity, assume that $V=\{1,2,\ldots,m+k\}$ with $k\geq 1$. Then

$$\begin{aligned}&\Pr(Y=1|X_S=x_S)\big|_{\mu=0,\bar{\beta}=0}\\&=\frac{1}{\Pr(X_S=x_S)}\sum_{x_{V\setminus S}}\Pr(X_V=x_V,Y=1)\big|_{\mu=0,\bar{\beta}=0}\\&=\sum_{x_{V\setminus S}}\Pr(X_{V\setminus S}=x_{V\setminus S})\sigma\left(\lambda\prod_{i\in V}x_i\right).\end{aligned}$$

Also by (5), we have

$$\begin{aligned}&\Pr(Y=1|X_S=x_S)\big|_{\mu=0,\bar{\beta}=0}\\&\quad=(A_{X_S=x_S}+C_{X_S=x_S})\sigma(\lambda)\\&\quad+(B_{X_S=x_S}+D_{X_S=x_S})\sigma(-\lambda).\end{aligned}$$

Thus,

$$\begin{aligned}E_{X_S=x_S}&=\sum_{x_{V\setminus S}:\ \prod_{i\in V}x_i=1}\Pr(X_{V\setminus S}=x_{V\setminus S})\\&\quad-\sum_{x_{V\setminus S}:\ \prod_{i\in V}x_i=-1}\Pr(X_{V\setminus S}=x_{V\setminus S})\\&=\sum_{x_{V\setminus S}:\ \prod_{i\in V\setminus S}x_i=\prod_{i\in S}x_i}\left(\prod_{i\in V\setminus S}p_{i,x_i}\right)\\&\quad-\sum_{x_{V\setminus S}:\ \prod_{i\in V\setminus S}x_i=-\prod_{i\in S}x_i}\left(\prod_{i\in V\setminus S}p_{i,x_i}\right)\\&=\prod_{i\in S}x_i\prod_{i\in V\setminus S}(p_i-q_i).\end{aligned}$$

Hence,

$$\begin{aligned}\sum_{x_S}\left(\prod_{i\in S}x_i\right)E_{X_S=x_S}&=\sum_{x_S}\prod_{i\in V\setminus S}(p_i-q_i)\\&=2^m\prod_{i\in V\setminus S}(p_i-q_i).\end{aligned}$$

By symmetry, if μ is β_S, there is

$$\sum_{x_S}\left(\prod_{i\in S}x_i\right)F_{x_S}=2^m;$$

if μ is β_V with $V\supset S$, there is

$$\sum_{x_S}\left(\prod_{i\in S}x_i\right)F_{x_S}=2^m\prod_{i\in V\setminus S}(p_i-q_i);$$

otherwise,

$$\sum_{x_S}\left(\prod_{i\in S}x_i\right)F_{x_S}=0.$$

The proof has been completed.

Proof of Equality (a) in (7)

We prove (a) by induction on $|S|$. When $|S|=1$,

$$\begin{aligned}&\sum_{\emptyset\subseteq T\subseteq\{1\}}(-1)^{1+|T|}\sum_{x_T}\frac{1}{\Pr(X_T=x_T)}\\&\quad\cdot\left(\sum_{x_{\{1\}\setminus T}}\Pr(X_1=x_1)\Lambda_{X_1=x_1}\right)^2\\&\quad=\frac{1}{p_1}(p_1\Lambda_{X_1=1})^2+\frac{1}{q_1}(q_1\Lambda_{X_1=-1})^2\\&\qquad-(p_1\Lambda_{X_1=1}+q_1\Lambda_{X_1=-1})^2\\&\quad=p_1q_1(\Lambda_{X_1=1}-\Lambda_{X_1=-1})^2\\&\quad=\prod_{i\in\{1\}}p_iq_i\left[\sum_{x_1}\left(\prod_{i\in\{1\}}x_i\right)\Lambda_{X_1=x_1}\right]^2.\end{aligned}$$

Assume (a) holds when $S=\{1,2,\ldots,k-1\}$. Now we consider the case when $S=\{1,2,\ldots,k\}$. Let $S'=S\setminus\{k\}$. Then

$$\begin{aligned}&\sum_{\emptyset\subseteq T\subseteq S}(-1)^{|S|+|T|}\sum_{x_T}\frac{1}{\Pr(X_T=x_T)}\\&\cdot\left(\sum_{x_{S\setminus T}}\Pr(X_S=x_S)\Lambda_{X_S=x_S}\right)^2\\&=\sum_{\emptyset\subseteq T\subseteq S'}(-1)^{|S|+|T|}\sum_{x_T}\frac{1}{\Pr(X_T=x_T)}\\&\cdot\left(\sum_{x_{S\setminus T}}\Pr(X_S=x_S)\Lambda_{X_S=x_S}\right)^2\\&+\sum_{\{k\}\subseteq T\subseteq S'\cup\{k\}}(-1)^{|S|+|T|}\sum_{x_T}\frac{1}{\Pr(X_T=x_T)}\\&\cdot\left(\sum_{x_{S\setminus T}}\Pr(X_S=x_S)\Lambda_{X_S=x_S}\right)^2\\&=\sum_{\emptyset\subseteq T'\subseteq S'}(-1)^{|S'|+1+|T'|}\sum_{x_{T'}}\frac{1}{\Pr(X_{T'}=x_{T'})}\\&\cdot\left(\sum_{x_{S'}}\Pr(X_{S'}=x_{S'})(p_k\Lambda_{X_{S'}=x_{S'},X_k=1}\right.\\&\left.+q_k\Lambda_{X_{S'}=x_{S'},X_k=-1})\right)^2+\sum_{\emptyset\subseteq T'\subseteq S'}(-1)^{|S'|+|T'|}\\&\cdot\left[\sum_{x_{T'}}\frac{1}{\Pr(X_{T'}=x_{T'})p_k}\left(\sum_{x_{S'\setminus T'}}\Pr(X_{S'}=x_{S'})p_k\right.\right.\\&\left.\cdot\Lambda_{X_{S'}=x_{S'},X_k=1}\right)^2+\sum_{x_{T'}}\frac{1}{\Pr(X_{T'}=x_{T'})q_k}\\&\left.\cdot\left(\sum_{x_{S'\setminus T'}}\Pr(X_{S'}=x_{S'})q_k\Lambda_{X_{S'}=x_{S'},X_k=-1}\right)^2\right]\\&=p_kq_k\sum_{\emptyset\subseteq T'\subseteq S'}(-1)^{|S'|+|T'|}\sum_{x_{T'}}\frac{1}{\Pr(X_{T'}=x_{T'})}\\&\cdot\left(\sum_{x_{S'\setminus T'}}\Pr(X_{S'}=x_{S'})(\Lambda_{X_{S'}=x_{S'},X_k=1}\right.\\&\left.-\Lambda_{X_{S'}=x_{S'},X_k=-1})\right)^2\\&\overset{(b)}{=}p_kq_k\cdot\left[\prod_{i\in S'}p_iq_i\right]\left[\sum_{x_{S'}}\left(\prod_{i\in S'}x_i\right)(\Lambda_{X_{S'}=x_{S'},X_k=1}\right.\\&\left.-\Lambda_{X_{S'}=x_{S'},X_k=-1})\right]^2\\&=\left[\prod_{i\in S}p_iq_i\right]\left[\sum_{x_S}\left(\prod_{i\in S}x_i\right)\Lambda_{X_S=x_S}\right]^2,\end{aligned}$$

where (b) follows by induction.

Proof of Theorem 5

Proof By Lemma 8, for any subset $\emptyset\subseteq T\subseteq S$,

$$\begin{aligned}|H(X_T,Y)-\mathbb{E}[\widehat{H}_N(X_T,Y)]|&\le\frac{2^{|T|+1}-1}{N}\\&<\frac{2^{|S|+1}}{2^{2|S|+2}/\delta}=\frac{\delta}{2^{|S|+1}}.\end{aligned}$$

Let $\widetilde{N}=\widetilde{N}(\varepsilon,\delta)$ for notational simplicity. With the facts that 1) $f(x)=\frac{x}{(\log x)^2}$ is strictly increasing over $[e^2,\infty)$ and 2) $x^{\frac{1}{e-2}}\ge\frac{e}{e-2}\log x$ for $x>0$ (both could be easily proved by the basic calculus), we have

$$\begin{aligned}\frac{N}{(\log N)^2}&\ge\frac{\widetilde{N}\left(\frac{e}{e-2}\log\widetilde{N}\right)^2}{\left(\log\widetilde{N}+2\log\frac{e}{e-2}+2\log\log\widetilde{N}\right)^2}\\&\ge\frac{\widetilde{N}\left(\frac{e}{e-2}\log\widetilde{N}\right)^2}{\left(\log\widetilde{N}+\frac{2}{e-2}\log\widetilde{N}\right)^2}=\widetilde{N}.\end{aligned}$$

Then by Lemma 8, we have

$$
\begin{aligned}
&\Pr\left(\left|\widehat{H}_N(X_T, Y) - H(X_T, Y)\right| > \frac{\delta}{2^{|S|}}\right) \\
&\leq \Pr\left(\left|\widehat{H}_N(X_T, Y) - \mathbb{E}[\widehat{H}_N(X_T, Y)]\right| > \frac{\delta}{2^{|S|+1}}\right) \\
&\leq 2\exp\left(-\frac{N}{2(\log N)^2} \cdot \frac{\delta^2}{2^{2|S|+2}}\right) \\
&\leq 2\exp\left(-\frac{\delta^2 \widetilde{N}}{2^{2|S|+3}}\right) = \frac{\varepsilon}{\max\left\{2^{|S|}, 3\right\}}.
\end{aligned}
$$

With a parallel argument for X_T with $|T| = 1$, we have

$$
\Pr\left(\left|\widehat{H}_N(X_T) - H(X_T)\right| > \frac{\delta}{2^{|S|}}\right) \leq \frac{\varepsilon}{\max\left\{2^{|S|}, 3\right\}}.
$$

Hence, we obtain

$$
\begin{aligned}
&\Pr\left(\left|\widehat{\mathscr{S}_N}(X_S; Y) - \mathscr{S}(X_S; Y)\right| > \delta\right) \\
&\leq \mathbf{1}_{|S|=1} \Pr\left(\left|\widehat{H}_N(X_S) - H_N(X_S)\right| > \frac{\delta}{2^{|S|}}\right) \\
&\quad + \sum_{\emptyset \subseteq T \subseteq S} \Pr\left(\left|\widehat{H}_N(X_T, Y) - H_N(X_T, Y)\right| > \frac{\delta}{2^{|S|}}\right) \\
&\leq \max\left\{2^{|S|}, 3\right\} \cdot \frac{\varepsilon}{\max\left\{2^{|S|}, 3\right\}} = \varepsilon.
\end{aligned}
$$

□
